# Tsaokoic Acid: A New Bicyclic Nonene from the Fruits of *Amomum tsao-ko* with Acetylcholinesterase Inhibitory Activity

**DOI:** 10.3390/molecules28062602

**Published:** 2023-03-13

**Authors:** Hyunyoung Kim, Hwaryeong Lee, Hee Jin Jung, Sang Gyun Noh, Isoo Youn, Hyunkyung Kwak, Yeju Lee, Sang-Jip Nam, Soosung Kang, Hae Young Chung, Eun Kyoung Seo

**Affiliations:** 1 Graduate School of Pharmaceutical Sciences, College of Pharmacy, Ewha Womans University, Seoul 03760, Republic of Korea; fanet0106@naver.com (H.K.); jongsky119@naver.com (H.L.); isooyoun87@gmail.com (I.Y.); yoohyun3581@naver.com (H.K.); rosac@ewhain.net (Y.L.); sskang@ewha.ac.kr (S.K.); 2 Department of Pharmacy, College of Pharmacy, Pusan National University, Busan 46241, Republic of Korea; hjjung2046@pusan.ac.kr (H.J.J.); rskrsk92@pusan.ac.kr (S.G.N.); hyjung@pusan.ac.kr (H.Y.C.); 3 Department of Chemistry and Nanoscience, Ewha Womans University, Seoul 03760, Republic of Korea; sjnam@ewha.ac.kr

**Keywords:** *Amomum tsao-ko*, Zingiberaceae, bicyclic nonene, acetylcholinesterase

## Abstract

A new bicyclic nonene, tsaokoic acid (**1**), was isolated from the fruits of *Amomum tsao-ko*, together with three known compounds (**2**–**4**). The structure of **1** was elucidated by analyzing spectroscopic data including 1D and 2D NMR spectra and compounds **2**–**4** were identified as tsaokoin, vanillin, and tsaokoarylone, respectively, by comparing their NMR spectra with previously reported data. Compounds **1**–**4** showed possible inhibitory activity against acetylcholinesterase (AChE) in silico molecular docking simulations. They were submitted to in vitro assay system and exhibited moderate inhibitory activity with IC_50_ values of 32.78, 41.70, 39.25, and 31.13 μM, respectively.

## 1. Introduction

*Amomum tsao-ko* Crevost et Lemair (Zingiberaceae) is a medicinal food known as “Cho-Gwa” in Korea and “Caoguo” or “Tsao-ko” in China [1]. The genus *Amomum* is distributed in tropical and subtropical regions of Asia and Oceania, and *A. tsao-ko* grows mainly in Southwestern China and Northern Vietnam [2,3]. The fruit of *A. tsao-ko* has been used as a spice and a traditional medicine in Asia to treat stomach disorders, dyspepsia, nausea, vomiting, diarrhea, malaria, throat infections, and abdominal pain [4,5,6]. It exhibits pharmacological activities such as anti-inflammatory, anti-tumor, anti-oxidant, anti-biotic, anti-diabetic, and neuroprotective effects [3,5,7]. Previous phytochemical studies on *A. tsao-ko* fruit isolated various natural compounds including flavonoids, terpenoids, and diarylheptanoids [2].

Alzheimer’s disease (AD) is a neurodegenerative disorder accompanied by clinical symptoms such as cognitive and language impairment, mental and behavioral difficulties, and problems with daily living activities. Although AD pathogenesis has not been clearly elucidated, one cause may be early loss of basal forebrain cholinergic neurons [8]. This explains the essential role of acetylcholine (ACh) in cognitive decline (including memory, attention, sensory, and learning) in AD [9]. Since cholinergic neurotransmission plays an important role in cognitive function, improving the acetylcholinesterase (AChE) brain level is crucial to treat AD. AChE is a cholinergic enzyme found primarily at neuromuscular junctions and chemical synapses of the cholinergic type responsible for terminating a synaptic transmission. Therefore, AChE inhibitors enhance cholinergic neurotransmission by increasing Ach levels [8,9].

**Figure 1:**
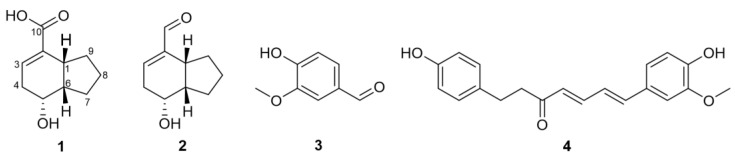
Chemical structures of isolates **1**–**4** from *A. tsao-ko*.

In this study, a new compound, tsaokoic acid (**1**), was isolated with three known compounds, tsaokoin (**2**), vanillin (**3**), and tsaokoarylone (**4**), from the *A. tsao-ko* fruits (Figure 1). **1**–**4** were tested for their AChE-inhibitory activity using an in silico molecular docking and an in vitro enzyme assay. This study describes their isolation, structure identification, and AChE-inhibitory activity.

## 2. Results and Discussion

### 2.1. Structure Elucidation

**Figure 2:**
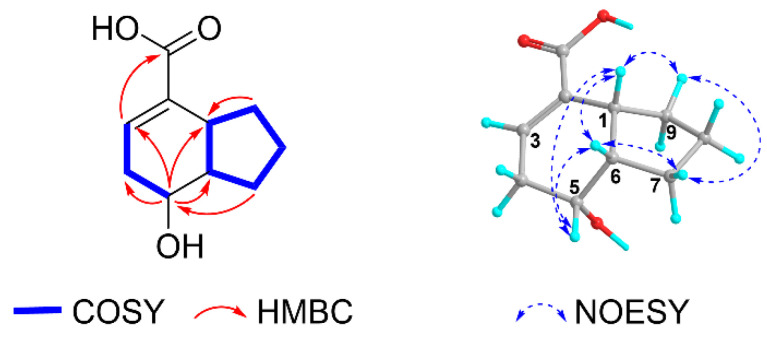
Key COSY, NOESY and HMBC correlations of **1**.

Compound **1** was obtained as white needles, and it exhibited a molecular ion peak at *m*/*z* 181.0868 [M − H]^−^ (calcd for 181.0870) in the HRESIMS, consistent with the molecular formula C_10_H_14_O_3_. The UV spectrum of **1** showed an absorption maximum at 240 nm. In the IR spectrum of **1**, a hydroxyl group and carboxylic acid functionality were observed at 2954 and 1685 cm^−1^, respectively. In the ^1^H and ^13^C NMR spectra of **1,** as shown in Table 1, four methylene functionalities appeared at *δ*_H_ 2.32 (1H, ddt, *J* = 17.8, 6.0, 1.5 Hz), 2.22 (1H, ddt, *J* = 18.0, 9.4, 2.9)/*δ*_C_ 30.8 (C-4), 1.72 (1H, dddd, *J* = 16.4, 8.4, 5.2, 4.1), 1.55 (1H, m)/26.0 (C-7), 1.55 (2H, m)/25.4 (C-8), and 2.03 (1H, dq, *J* = 5.8, 2.4), 1.45 (1H, m)/34.2 (C-9). Four methines were also observed at *δ*_H_ 6.78 (1H, dq, *J* = 6.0, 1.5 Hz)/*δ*_C_ 136.5 (C-3), 3.94 (1H, dt, *J* = 9.6, 4.8)/68.9 (C-5), 3.01 (1H, ddt, *J* = 9.8, 3.3, 1.6)/40.9 (C-1), and 2.41 (1H, dddd, *J* = 12.6, 8.0, 4.0, 1.4)/44.6 (C-6) together with a quaternary carbon peak at *δ*_C_ 135.9 (C-2), indicating the presence of a bicyclic nonene [1]. In the HMBC spectrum of **1**, the bicyclic nonene group was confirmed by the correlations of H-1/C-2, C-9, H-3/C-1, C-2, C-4, C-5, H-4/C-2, C-3, C-5, C-6, H-6/C-1, C-2, C-4, C-5, C-7, H-7/C-1, C-5, C-6, C-8, C-9, H-8/C-1, C-6, C-9, and H-9/C-1, C-2, C-6, C-7, C-8. In addition, the ^13^C NMR resonance at *δ*_C_ 170.7 (C-10) displayed a carboxylic acid in **1**. A hydroxyl group was expected from molecular ion peak of HRESIMS. The carboxylic acid was positioned at C-2 due to the HMBC correlation between the proton H-3 at *δ*_H_ 6.78 and the carboxylic carbon at *δ*_C_ 170.7 (C-10) (Figure 2) which are three bonds away from each other. The hydroxyl group was assigned at C-5 (*δ*_C_ 68.9) as a secondary hydroxyl since the H-5 at *δ*_H_ 3.94 showed two-bond correlations with C-4 and C-6 and three-bond connectivities with C-1, C-3, and C-7 in the HMBC NMR spectrum of **1**. As a result, the structure of **1** was similar to tsaokoin (**2**) [1], except for the carboxylic acid in **1** instead of an aldehyde group in tsaokoin (**2**) at C-10. 

The relative configuration of **1** was determined by analyzing its NOESY spectrum (Figure 2). The NOE correlations of H-1/H-5, H-1/H-6, and H-5/H-6 indicated that the three protons of H-1, H-5, H-6 are cofacial, while a hydroxyl group at C-5 is on the opposite side. Therefore, the structure of **1** was determined as a new compound, rel-(1*R*,5*R*,6*S*)-5-hydroxybicyclo[4.3.0]non-2-ene-2-carboxylic acid (Figure 1), namely, “tsaokoic acid”. 

Compound **2** was isolated as a colorless oil and its molecular formula determined as C_10_H_14_O_2_ based on the HRESIMS ([M + H]^+^, *m*/*z* 167.1072, calcd for 167.1067). The ^1^H and ^13^C chemical shifts (Table 1) of **2** also exhibited peak values for bicyclic nonene fragments that were identical to those of tsaokoin [1,10]. ^13^C NMR resonances at C-2 (*δ*_C_ 144.9) and C-3(*δ*_C_ 146.5) showed larger chemical shifts compared to those of **1**. Unlike **1**, *δ*_H_ 9.42 (s)/*δ*_C_ 193.8 (C-10) showed the presence of carbaldehyde group instead of carboxylic group at C-2 position, which was supported by the HMBC correlations between H-10/C-1, C-2, and C-3 and NOESY correlations of H-3/H-10. The relative configuration of **2** was confirmed as 1*R*,5*R*,6*S*, which is the same as compound **1** based on the NOE correlations of H-1/H-5, H-5/H-6, and H-1/H-6 in compound **2**. Thus, **2** was identified as the known compound, rel-(1*R*,5*R*,6*S*)-5-hydroxybicyclo[4,3,0]non-2-ene-2-carboxaldehyde (Figure 1) [10,11].

Relative configurations for **1** and **2**, were determined according to their NOESY data as described in the results. Compounds **1** and **2** have the same relative configurations as they showed identical NOESY correlations of H-1/H5, H-1/H-6, and H-5/H-6, which indicate that the three protons of H-1, H-5, and H-6 are in *cis* configuration with each other. To determine their absolute configurations, Mosher’s esterification experiments were performed for **1** and **2.** However, the results showed identical ^1^H NMR spectra of (*R*)- and (*S*)-MTPA esters, indicating racemic mixtures. The optical rotation values of **1** and **2** were [α]^22^_D_ −1.65 (*c* 0.1, MeOH) and [α]^20^_D_ −0.96 (*c* 0.1, CH_2_Cl_2_), respectively, which indicates that they were not perfectly racemic. However, we were unable to find any differences in chemical shifts in the ^1^H NMR spectra of (*R*)- and (*S*)-MTPA esters of compounds **1** and **2**. Thus, we think that **1** and **2** are racemic mixtures. All figures, including 1D and 2D NMR spectra of compounds **1** and **2**, and the ^1^H NMR data for MPTA esters of compounds **1** and **2**, were provided in the supplementary materials (Figures S1–S24).

### 2.2. AChE-Inhibitory Activities of **1**–**4**

In the present study, compounds **1**–**4** showed possible inhibitory activity against AChE in molecular docking simulations, and thus, in vitro assays have been performed against AChE and more details are as follows.

#### 2.2.1. In Silico Docking Simulation

**Figure 3:**
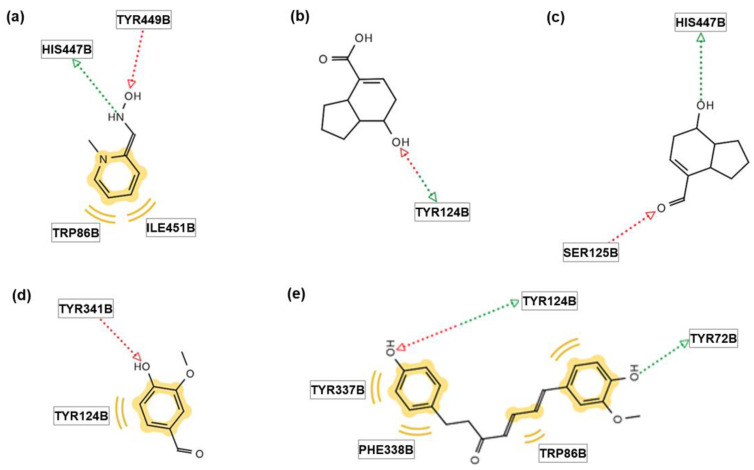
The pharmacophores of the tested compounds indicate the interactions between the receptor (AChE) and ligands (**1**–**4** and FP1). The green and red arrows indicate the hydrogen bond donor and acceptor, respectively. The yellow color indicates a hydrophobic interaction or van der Waals Force. (**a**) FP1, a positive control, (**b**) **1**, (**c**) **2**, (**d**) **3**, and (**e**) **4**.

Before the in vitro anti-AChE activity test, an in silico docking simulation was performed to predict the specific pharmacological effects of the four compounds (**1**–**4**) against AChE. The binding sites in the enzyme and binding energies of each compound were predicted through a docking simulation utilizing three systems (Autodock vina, Autodock 4, LeDock). Figure 3 shows the binding sites of the AChE receptor and ligands. FP1, the positive control, had two hydrogen bonds and two hydrophobic interactions with AChE (Figure 3a). The binding affinity of the tested compounds was compared to those of the control (FP1) for hydrogen bonding and hydrophobic interaction. Tsaokoic acid (**1**) and tsaokoin (**2**) did not have hydrophobic interactions with the enzyme and thus showed lower binding affinities than the control (FP1) (Figure 3b,c). Vanillin (**3**) possessed one hydrogen bond and one hydrophobic interaction with AChE, showing a lower binding force than the control (Figure 3d). On the other hand, tsaokoarylone (**4**) showed a stronger binding affinity than the control (FP1) as four hydrophobic interactions and two hydrogen bonds were observed (Figure 3e).

The docking scores of the receptor and compounds are shown in Table 2. Higher absolute values indicate a stronger binding affinity. A control group (FP1) showed −5.6, −5.11, and −2.52 for the Autodock Vina, Autodock 4, and LeDock systems, respectively. Tsaokoic acid (**1**), tsaokoin (**2**), and vanillin (**3**) showed scores to AChE with values of −5.8~−6.8 (Autodock Vina), −5.17~−6.07 (Autodock 4), and −2.7~−3.32 (LeDock), which were higher than those of FP1. On the other hand, tsaokoarylone (**4**) showed the highest docking scores, −7.2, −8.58, and −4.1, which corresponded to the docking simulation results. All the docking simulations were repeated three times.

#### 2.2.2. In Vitro Assay for AChE-Inhibitory Activity

**Figure 4:**
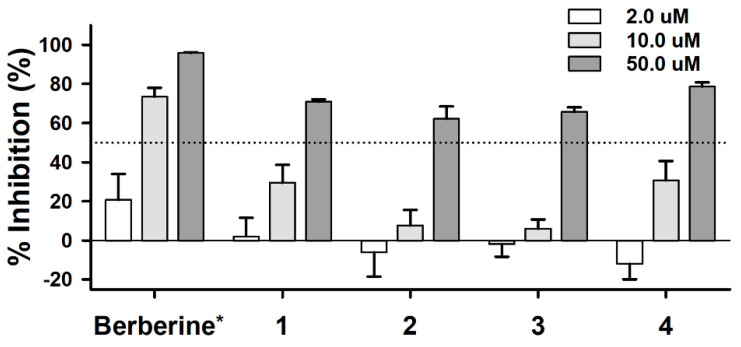
AChE-inhibitory activity (%) of the four compounds (**1**–**4**) at three concentrations by five-fold dilution. * Concentrations of the control (berberine) were 0.04, 0.2, and 1.0 µM, respectively.

On the basis of the results from the in silico docking simulation for AChE-inhibitory activity, **1**–**4** were tested in vitro screening system at total concentrations of 2, 10, and 50 μM and berberine was used as a positive control (Figure 4 and Table S1). The half-maximal inhibitory concentrations (IC_50_) of **1**–**4** were 32.78, 41.70, 39.25, and 31.13 μM, respectively, as shown in Figure 4. These results can be considered as moderate AChE-inhibitory activity compared to the positive control, berberine (IC_50_ 0.19 μM). Isolates **1**–**4** showed consistent activity between in silico and in vitro experiments. Therefore, we can utilize this in silico docking simulation system to find any possible AChE inhibitors before we perform the in vitro or in vivo experiments to save our time and expenses. This is the first report on the AChE-inhibitory activities of compounds **1**, **2**, and **4**. Compound **2**, tsaokoin, was reported to have weak antifungal activity in previous studies, but its AChE-inhibitory activity has not been reported [10]. Previous in vitro and in vivo reports indicate that compound **3** has AChE-inhibitory activities [12,13,14]. In this study, in silico docking experiments on AChE for vanillin (**3**) were performed for the first time as well as for compounds **1**, **2**, and **4**. This study is considered a good example of in silico research that aligns with in vitro results. Their AChE-inhibitory activity indicates that compounds **1**–**4** have some possibilities to treat mild Alzheimer’s disease, by increasing the level of ACh.

## 3. Materials and Methods

### 3.1. General Experimental Procedures

Optical rotation data were obtained on a JASCO P-2000 polarimeter (Tokyo, Japan). UV spectra were measured on a Hitachi U-3000 UV/Vis spectrophotometer (Tokyo, Japan). IR spectra were recorded on a Thermo Fisher Nicolet iS 10 FT-IR spectrometer (Waltham, MA, USA). NMR spectra were acquired on an Agilent DD2 400 MHz FT-NMR instrument (Agilent Technologies, Santa Clara, CA, USA) using tetramethylsilane as an internal standard and analyzed with MestreNova 9.0.0 software (Mestrelab Research S.L., Santiago de Compostela, Spain). HRESIMS was performed on an Agilent 6230 TOF LC/MS instrument (Agilent Technologies, Santa Clara, CA, USA) equipped at Ewha Drug Development Research Core Center. Adsorption column chromatography was conducted using silica gel (63–200 μm, Merck, Darmstadt, Germany). MPLC was run on a CombiFlash Rf-200 instrument (Teledyne Isco, Lincoln, NE, USA) and RediSep^®^ Silver Silica Gel Disposable Flash Columns 330.0 g and 24.0 g (Teledyne Isco, Lincoln, NE, USA) were used for separations. The Acme 9000 system (Young Lin, Anyang-si, Gyeonggi-do, Republic of Korea) with UV detection was used for analytic HPLC, equipped with an Agilent Prep-C18 Scalar column (4.6 × 250 mm, 5 µm, Santa Clara, CA, USA). Preparative HPLC was carried out on a YMC-Pack Pro C 18 column (20 × 250 mm, 5 µm, Asan-si, Chungcheongnam-do, Republic of Korea) using a Waters system equipped with a Waters 600 pump and a Waters 996 photodiode array detector (Waters, MA, USA). Thin-layer chromatography (TLC) was conducted using Kieselgel 60 F_254_ aluminum sheets (Merck, Darmstadt, Germany) and RP-18 F_254s_ aluminum sheets (Merck, Darmstadt, Germany). TLC plates were visualized under UV (254 and 365 nm) after being dipped in a 10% (*v*/*v*) sulfuric acid solution and heated at 300 °C for 1 min. The solvents used for HPLC experiments were HPLC-grade (Daejung Chemicals & Metals, Siheung-si, Gyeonggi-do, Republic of Korea). Solvents for NMR experiments were purchased from Cambridge Isotope Laboratories (Tewksbury, MA, USA).

### 3.2. Plant Material

The dried fruits of *Amomum tsaoko* Crevost et Lemaire (Zingiberaceae) were purchased from Nonglim Saengyak Company (Agricultural and Forestry Herb Market) in Seoul, South Korea in June 2020. A voucher specimen (no. EA389) was deposited at the Natural Product Chemistry Laboratory, College of Pharmacy, Ewha Womans University.

### 3.3. Extraction and Isolation

The dried fruits of *A. tsaoko* (10.0 kg) were extracted three times with 32 L of MeOH at room temperature over a period of one week each time. The extract was dried under reduced pressure to obtain 606.0 g of a MeOH concentrate. After dissolving the concentrate in distilled water (1 L), the mixture was sequentially fractionated with *n*-hexane (10 × 1 L), EtOAc (12 × 1 L), and *n*-BuOH (10 × 1 L) to afford *n*-hexane-soluble (112.0 g), EtOAc-soluble (144.0 g), *n*-BuOH-soluble concentrate (132.0 g), and aqueous residue (216.0 g), respectively. The EtOAc fraction (144.0 g) was applied to a silica gel column chromatography using a gradient solvent system of CH_2_Cl_2_-MeOH (100:0 to 0:100, *v*/*v*) to obtain nine fractions (F01–F09). Fraction F02 (2.3 g) was subjected to MPLC with a gradient mixture of *n*-hexane-EtOAc (100:0 to 0:100, *v*/*v*) successively to provide **2** (26 mg) with eight subfractions (F02.15.01–F02.15.08). Fraction F03 (8.1 g) was subjected to MPLC with a gradient solvent system (*n*-hexane-EtOAc, 90:10 to 0:100, *v*/*v*) to yield 14 subfractions (F03.01–F03.14). Fraction F03.07 (744 mg) was loaded on successive MPLC separations, and then subfraction F03.07.03.02 was purified using preparative HPLC with MeOH-H_2_O (60:40, 2 mL/min) to obtain **3** (3 mg; *t*_R_ 26 min). Fraction F03.08 (1.5 g) was separated by MPLC using *n*-hexane-acetone (100:0 to 0:100, *v*/*v*) to give six subfractions (F03.08.01–F.03.08.06) and fraction F03.08.02 (1.0 g) was separated using *n*-hexane-acetone (100:0 to 70:30, *v*/*v*) to obtain six subfractions (F03.08.02.01–F.03.08.02.06). Fraction F03.08.02.05 (89 mg) was further purified by preparative HPLC eluting with an isocratic mixture of MeOH-H_2_O (60:40, 2 mL/min) to isolate **1** (30 mg; *t*_R_ 15 min). Fraction F03.10 (143 mg) was fractionated by MPLC with *n*-hexane-acetone (100:0 to 70:30 *v*/*v*) to afford four subfractions (F03.10.01–F03.10.04). Fraction F03.10.02 (20 mg) was loaded on the HPLC using MeOH-H_2_O (60:40, 2 mL/min) to purify **4** (5 mg; *t*_R_ 20 min).

Tsaokoic acid (**1**): White needles; [α]^22^_D_ −1.65 (*c* 0.1, MeOH); UV (MeOH) *λ*_max_ (log ε) 240 (2.72) nm; IR (KBr) *ν*_max_ 2954, 2868, 1685, 1638, 1249, 1054 cm^−1^; ^1^H NMR (CD_3_OD, 400 MHz) and ^13^C NMR (CD_3_OD, 100 MHz) data, see Table 1; HRESIMS *m*/*z* 181.0868 [M − H]^−^ (calcd for C_10_H_14_O_3_, 181.0870).

Tsaokoin (**2**): Colorless oil; [α]^20^_D_ −0.96 (*c* 0.1, CH_2_Cl_2_); UV (CH_2_Cl_2_) *λ*_max_ (log ε) 230 (2.40) nm; IR (KBr) *ν*_max_ 3418, 2955, 2869, 2722, 1681, 1635, 1451, 1430, 1378, 1309, 1167, 1115, 1061, 1025, 968, 926, 902, 827, 739, 585 cm^−1^; ^1^H NMR (CDCl_3_, 400 MHz) and ^13^C NMR (CDCl_3_, 100 MHz) data, see Table 1; HRESIMS *m*/*z* 167.1072 [M + H]^+^ (calcd for C_10_H_14_O_2_, 167.1067).

Vanillin (**3**): White powder; ^1^H NMR and ^13^C NMR data were comparable to the reference data [15].

Tsaokoarylone (**4**): Yellowish amorphous solid; ^1^H NMR and ^13^C NMR data were comparable to the reference data [16].

### 3.4. In Silico AChE-Inhibitory Activity

For the docking studies, the crystal structure of AChE was obtained from the RCSC PDB website (PDB ID: 5HFA) (https://www.rcsb.org/, accessed on 20 September 2022). The 3D structures of tsaokoic acid, tsaokoin, and tsaokoarylone were built by ACD/ChemSketch freeware (ACD/Labs, Toronto, ON, Canada) (https://www.acdlabs.com/resources/free-chemistry-software-apps/chemsketch-freeware/, accessed on 20 September 2022), while the 3D structure of vanillin was obtained from the Pubchem website (https://pubchem.ncbi.nlm.nih.gov/, accessed on 20 September 2022). Three programs were used for docking simulation: Autodock Vina 1.1.2 (Scripps Research, San Diego, CA, USA) (https://vina.scripps.edu/, accessed on 20 September 2022), Autodock4.2.6 (Scripps Research, San Diego, CA, USA) (https://autodock.scripps.edu/, accessed on 20 September 2022), and LeDock (LEPHAR, http://www.lephar.com/software.htm, accessed on 20 September 2022). Docking preparation of four compounds was conducted by UCSF Chimera program (University of California, San Francisco, CA, USA) (https://www.rbvi.ucsf.edu/chimera/, accessed on September 2022). A pharmacophore analysis between AChE and the compounds was conducted by LigandScout 4.0 (inte:ligand, Maria Enzersdorf, Niedaestareich, Austria) (http://www.inteligand.com/ligandscout/, accessed on 20 September 2022).

### 3.5. In Vitro AChE-Inhibitory Assay

The inhibitory activities of the compounds on AChE were measured using the spectrophotometric method developed by Ellman et al. 1961 [17]. The reaction mixtures contained 140 µL of sodium phosphate buffer (pH 8.0), 20 µL of tested sample solution, and 20 µL of AChE solution, which were mixed and incubated for 15 min at room temperature. All tested compounds and positive control (berberine) were dissolved in 10% DMSO Reactions were initiated with the addition of 10 µL of dithiobisnitrobenzoic acid (DTNB) and 10 µL of ACh. The hydrolysis of ACh was monitored by tracking the formation of 5-thio-2-nitrobenzoate anion at 412 nm for 15 min, resulting from the reaction of DTNB with the thiocholine released by the enzyme. Each reaction was performed in triplicate and the results were measured in 96-well microplates using a microplate spectrophotometer (Tecan, Sunrise, Austria). Percent inhibition was calculated using the formula (1 − S/E) × 100, where E and S are enzyme activities with and without the test sample, respectively. The inhibitory activity of each compound against AChE was expressed as an IC_50_ (the µM concentration required to inhibit substrate hydrolysis by 50%), as calculated using log-dose inhibition curves.

## 4. Conclusions

In this study, tsaokoic acid (**1**), tsaokoin (**2**), vanillin (**3**), and tsaokoarylone (**4**) were isolated from the EtOAc fraction of the *A. tsao-ko* fruits. Compound **1** was elucidated as a new compound, tsaokoic acid, using various spectroscopic data including 1D and 2D NMR techniques such as COSY, NOESY, HSQC, and HMBC NMR experiments. Relative configurations for **1** and **2** were determined according to their NOESY data as described in the results. Compounds **1**–**4** exhibited moderate AChE-inhibitory activities at IC_50_ values of 32.78, 41.70, 39.25, and 31.13 μM, respectively, which are consistent with the results of in silico docking simulations. From these results, we can conclude that in silico docking simulation system for AChE-inhibitory activiy could be a guide for in vitro system. Compounds **1**–**4** isolated from the fruits of *A. tsao-ko,* have some possibilities to enhance cognition in humans, treating mild Alzheimer’s disease.

## Figures and Tables

**Table 1:** ^1^H (400 MHz) and ^13^C (100 MHz) NMR data for **1** and **2**.

Position	1 ^a^			2 ^b^		
	*δ*_C_	Type	*δ*_H_ (*J* in Hz)	*δ*_C_	Type	*δ*_H_ (*J* in Hz)
1	40.9	CH	3.01 ddt (9.8, 3.3, 1.6)	37.3	CH	2.98 m
2	135.9	C		144.9	C	
3	136.5	CH	6.78 dq (6.0, 1.5)	146.5	CH	6.64 ddd (5.6, 3.2, 1.3)
4	30.8	CH_2_	2.32 ddt (17.8, 6.0, 1.5)	31.2	CH_2_	2.53 dtt (18.4, 5.2, 1.5)
			2.22 ddt (18.0, 9.4, 2.9)			2.40 td (8.6, 2.9)
5	68.9	CH	3.94 dt (9.6, 4.8)	68.4	CH	4.05 dt (8.8, 4.8)
6	44.6	CH	2.41 dddd (12.6, 8.0, 4.0, 1.4)	42.8	CH	2.44 dt (8.8, 2.8)
7	26.0	CH_2_	1.72 dddd (16.4, 8.4, 5.2, 4.1)	25.0	CH_2_	1.78 td (8.8, 5.2)
			1.55 m			1.63 m
8	25.4	CH_2_	1.55 m	24.7	CH_2_	1.56 ddd (15.6, 7.2, 1.9)
9	34.2	CH_2_	2.03 dq (5.6, 2.4)	32.3	CH_2_	2.12 ddd (20.8, 7.6, 5.1)
			1.45 m			1.41 tdd (13.2, 5.6, 1.7)
10	170.7	COOH		193.8	CHO	9.42s

^a^ Data were measured in CD_3_OD. ^b^ Data were measured in CDCl_3_.

**Table 2:** In silico docking scores of the control and the compounds **1**–**4** against AChE.

Compound	Autodock Vina ^a^	Autodock4 ^a^	LeDock ^a^	No. of H-Bond	H-Bond Interacting Residues	Hydrophobic Interacting Residues
FP1 ^b^	−5.6	−5.11	−2.52	2	HIS447B, TYR449B	ILE451B, TRP86B
**1**	−6.8	−5.24	−3.32	1	TYR124B	
**2**	−6.3	−6.07	−2.93	2	HIS447B, SER125B	
**3**	−5.8	−5.17	−2.7	1	TYR341B	TYR124B
**4**	−7.2	−8.58	−4.1	2	TYR72B, TYR124B	PHE338B, TRP86B, TYR337B

^a^ Unit: Kcal/mol, ^b^ Control.

## Data Availability

Not applicable.

## Supplementary Materials

The following supporting information can be downloaded at: https://www.mdpi.com/article/10.3390/molecules28062602/s1. Figures S1–S7 with 1D/2D NMR spectroscopic data of compound 1; Figures S8–S10 with UV, IR, HRESIMS data of compound **1**; Figures S11–S17 with 1D/2D NMR spectroscopic data of compound 2; Figures S18–S20 with UV, IR, HRESIMS data of compound **2**; Figures S21–S24 with 1H-NMR data of MTPA esters of **1** and **2**; Table S1 with AChE-inhibitory activity of the isolates **1**–**4**.
