# Supplementary material for: Tsaokoic Acid: A New Bicyclic Nonene from the Fruits of Amomum tsao-ko with Acetylcholinesterase Inhibitory Activity
Source: Molecules. 2023 Mar 13;28(6):2602. doi: 10.3390/molecules28062602 (PMC10059965; doi:10.3390/molecules28062602)
Supplement: Supplementary file 1 [file molecules-28-02602-s001.zip › molecules-2266567-supplementary.pdf]

Supplementary Materials

Tsaokoic acid: a new bicyclic nonene from the fruits of *Amomum tsao-ko* with acetylcholinesterase inhibitory activity

Hyunyoung Kim [1,#], Hwaryeong Lee [1,#], Hee Jin Jung [3], Sang Gyun Noh [3], Isoo Youn [1], Hyunkyung Kwak [1], Yeju Lee [1], Sang-Jip Nam [2], Soosung Kang [1], Hae Young Chung [3] and Eun Kyoung Seo [1,*]

[1]Graduate School of Pharmaceutical Sciences, College of Pharmacy, Ewha Womans University, Seoul 03760, Korea

[2]Department of Chemistry and Nanoscience, Ewha Womans University, Seoul 03760, Korea

[3]Department of Pharmacy, College of Pharmacy, Pusan National University, Busan 46241, Korea

[*]Correspondence: yuny@ewha.ac.kr (E.K.S.)

[#]Equally contributed in this study.

Contents

Table S1. AChE-inhibitory activity of the isolates **1-4** from *A. tsao-ko* -------------------- 4

Figure S1. ^{1}H NMR spectrum of **1** (400 MHz, CD_3OD) ------------------------------------- 5

Figure S2. ^{13}C NMR spectrum of **1** (100 MHz, CD_3OD) ------------------------------------ 5

Figure S3. DEPT-135 NMR spectrum of **1** (100 MHz, CD_3OD) --------------------------- 6

Figure S4. ^{1}H-^{13}C HSQC NMR spectrum of **1** (CD_3OD) ------------------------------------ 6

Figure S5. ^{1}H-^{13}C HMBC NMR spectrum of **1** (CD_3OD) ----------------------------------- 7

Figure S6. ^{1}H-^{1}H COSY NMR spectrum of **1** (CD_3OD) ------------------------------------- 7

Figure S7. ^{1}H-^{1}H NOESY NMR spectrum of **1** (CD_3OD) ----------------------------------- 8

Figure S8. UV spectrum of **1** -- 8

Figure S9. IR spectrum of **1**--- 9

Figure S10. HRESIMS spectrum of **1** --- 9

Figure S11. ^{1}H NMR spectrum of **2** (400 MHz, $CDCl_3$) ------------------------------------- 10

Figure S12. ^{13}C NMR spectrum of **2** (100 MHz, $CDCl_3$) ------------------------------------ 10

Figure S13. DEPT-135 NMR spectrum of **2** (100 MHz, $CDCl_3$) --------------------------- 11

Figure S14. ^{1}H-^{13}C HSQC NMR spectrum of **2** ($CDCl_3$) ------------------------------------ 11

Figure S15. ^{1}H-^{13}C HMBC NMR spectrum of **2** ($CDCl_3$) ----------------------------------- 12

Figure S16. ^{1}H-^{1}H COSY NMR spectrum of **2** ($CDCl_3$) ------------------------------------- 12

Figure S17. ^{1}H-^{1}H NOESY NMR spectrum of **2** ($CDCl_3$) ----------------------------------- 13

Figure S18. UV spectrum of **2** -- 13

Figure S19. IR spectrum of **2** --- 14

Figure S20. HRESIMS spectrum of **2** -- 14

Figure S21. ^{1}H -NMR (400 MHz, CD_3OD) of (R)-MTPA ester of **1** ------------------------- 15

Figure S22. ^{1}H -NMR (400 MHz, CD_3OD) of (S)-MTPA ester of **1** ------------------------- 15

Figure S23. ^{1}H -NMR (400 MHz, $CDCl_3$) of (R)-MTPA ester of **2** -------------------------- 16

Figure S24. ^{1}H -NMR (400 MHz, $CDCl_3$) of (S)-MTPA ester of **2** -------------------------- 16

	Concentration (μM)	Inhibition (%)	IC_{50} (μM)	sd[a]
	2	2.09	32.78	1.00
tsaokoic acid (**1**)	10	29.50		
	50	70.92		
	2	-6.07	41.70	3.19
Tsaokoin (**2**)	10	7.74		
	50	62.13		
	2	-1.88	39.25	1.81
Vanillin (**3**)	10	6.07		
	50	65.69		
	2	-11.93	31.13	1.24
Tsaokoarylone (**4**)	10	30.75		
	50	78.66		
	0.04	20.71	0.19	0.09
Berberine	0.2	73.43		
	1	95.82		

[a]Standard deviation

Table S1. AChE-inhibitory activity of the isolates **1-4** from *A. tsao-ko*

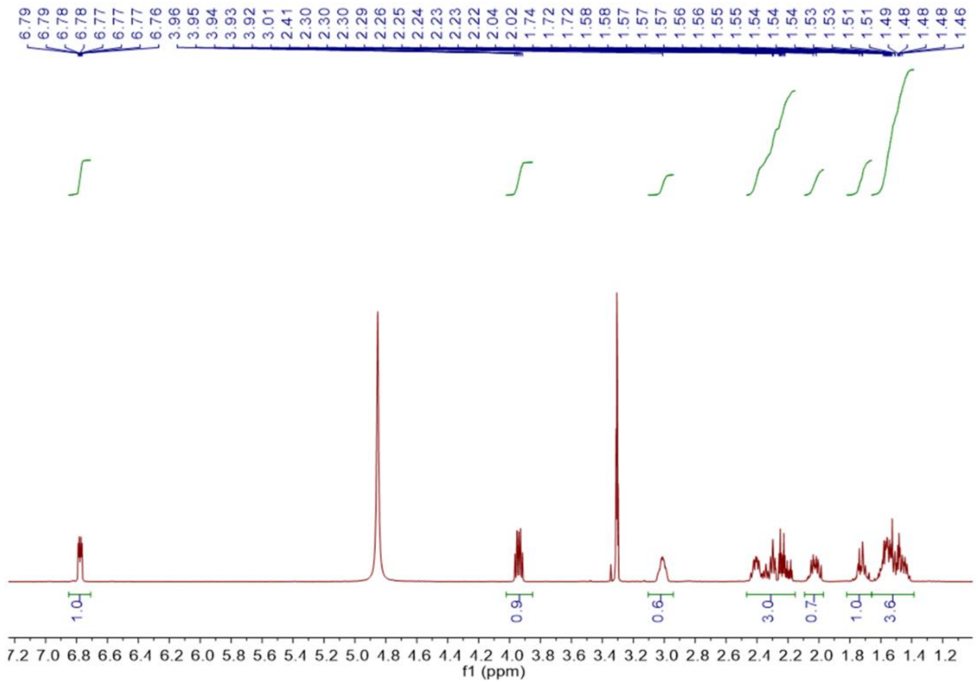

Figure S1. ^{1}H NMR spectrum of **1** (400 MHz, CD_3OD)

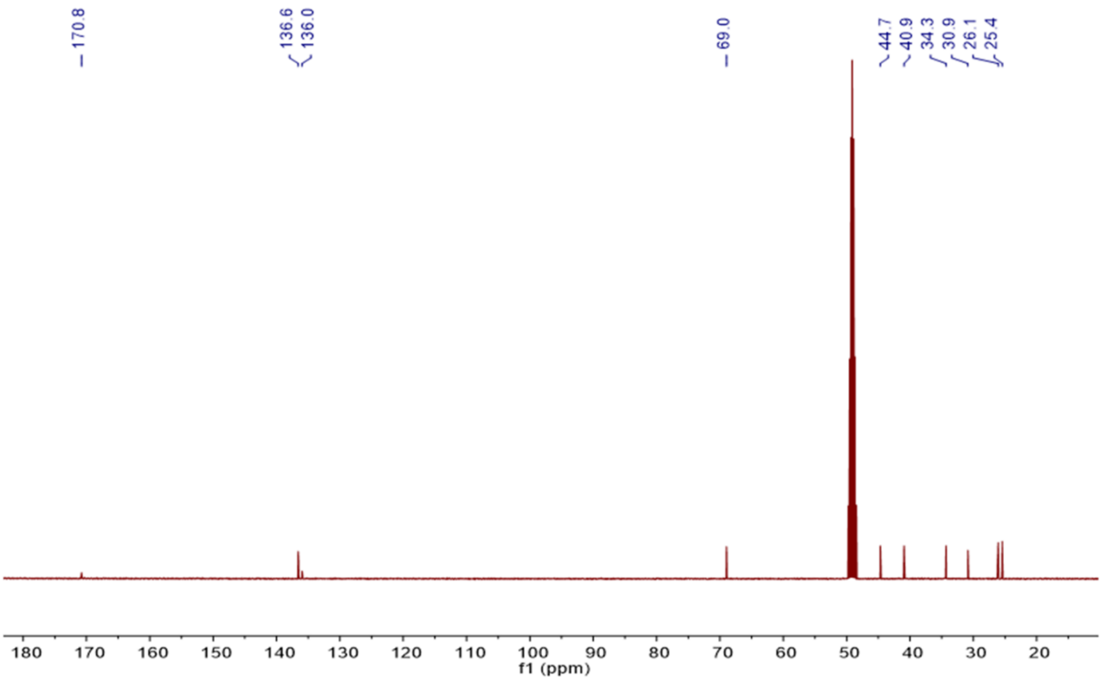

Figure S2. 13C NMR spectrum of **1** (100 MHz, CD_3OD)

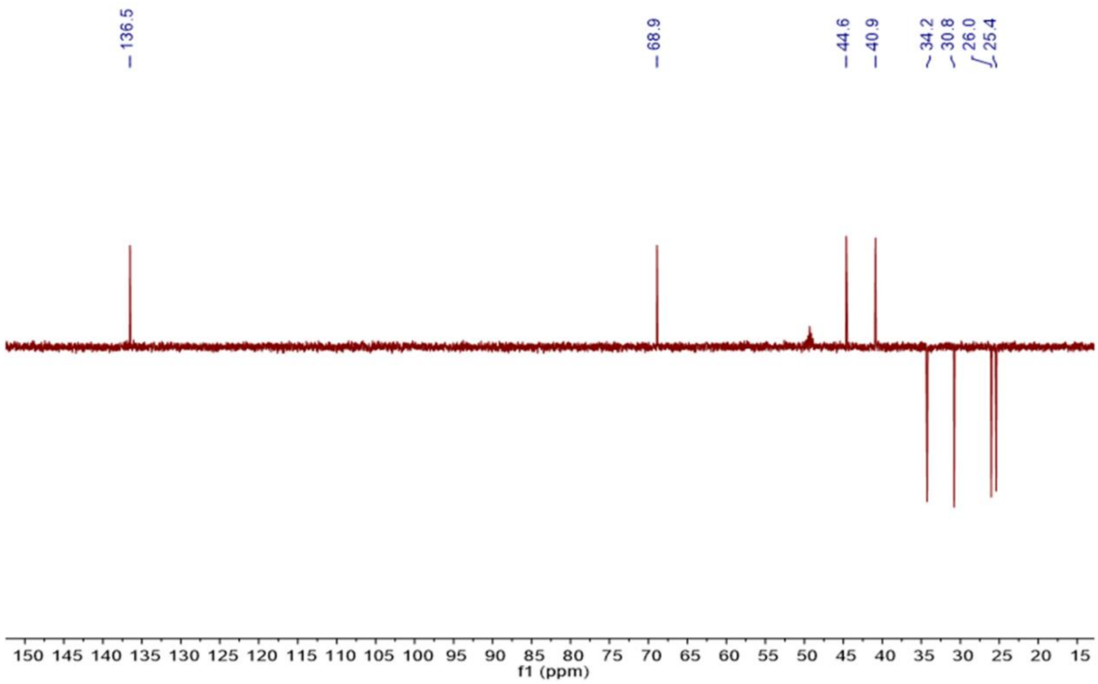

Figure S3. DEPT-135 NMR spectrum of **1** (100 MHz, CD_3OD)

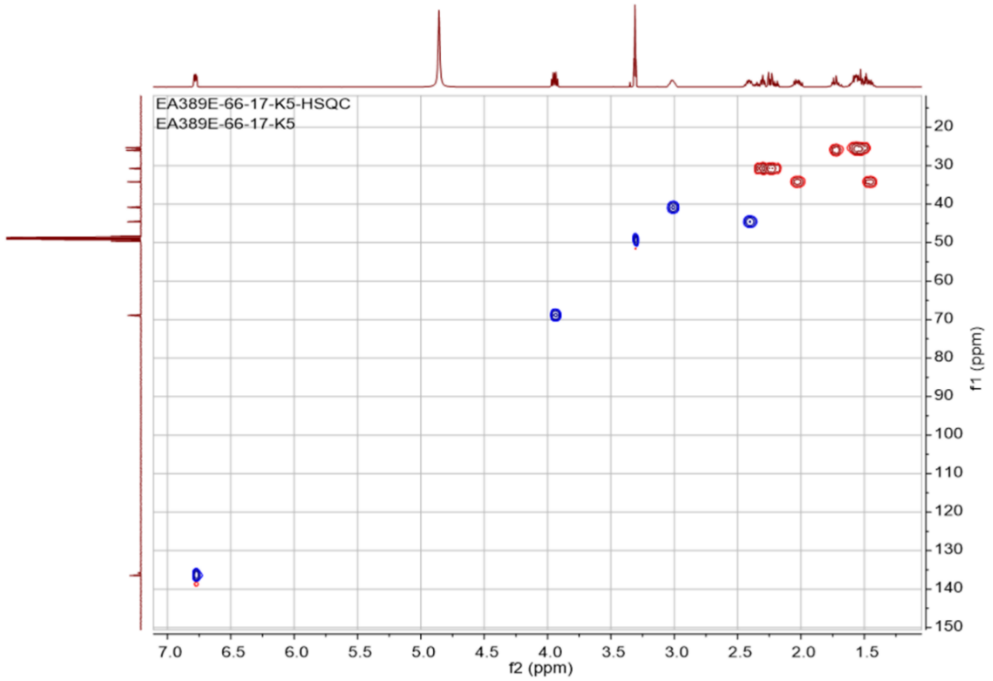

Figure S4. 1H-13C HSQC NMR spectrum of **1** (CD_3OD)

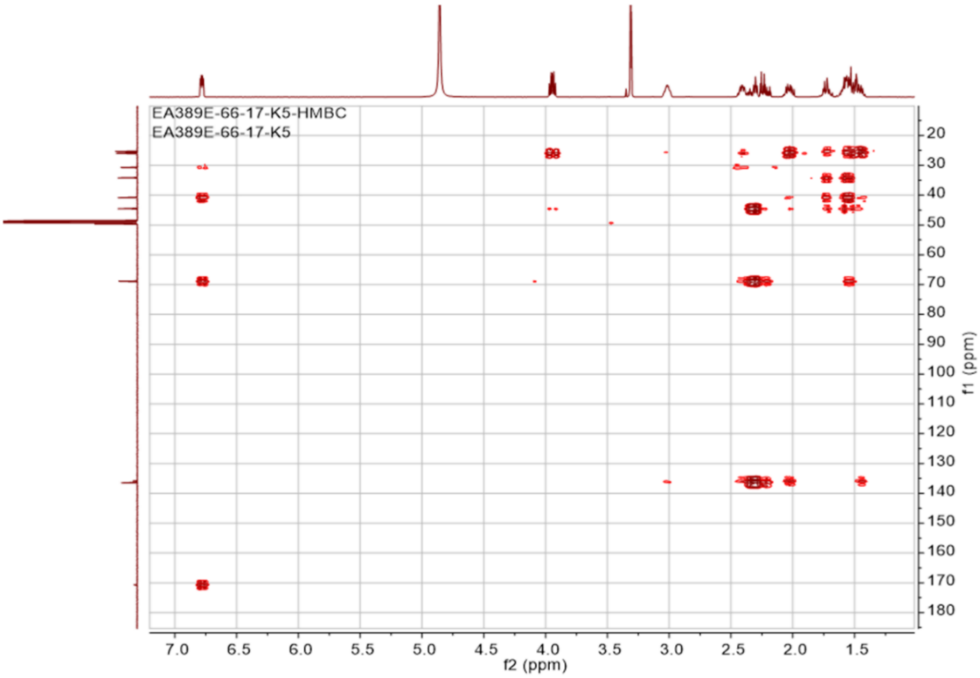

Figure S5. 1H-13C HMBC NMR spectrum of **1** (CD_3OD)

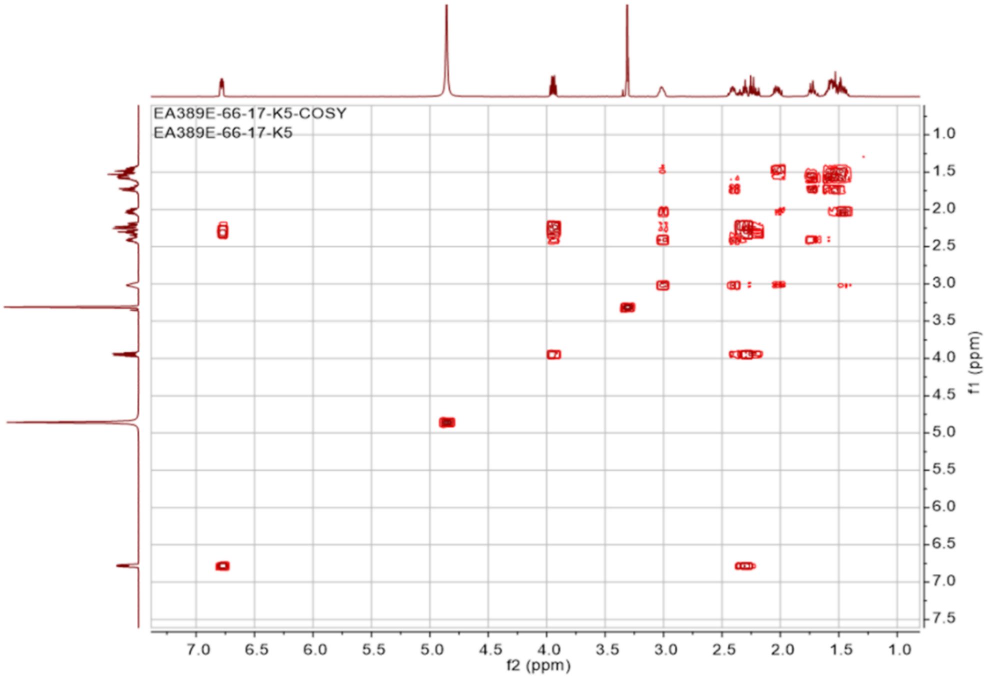

Figure S6. 1H-1H COSY NMR spectrum of **1** (CD_3OD)

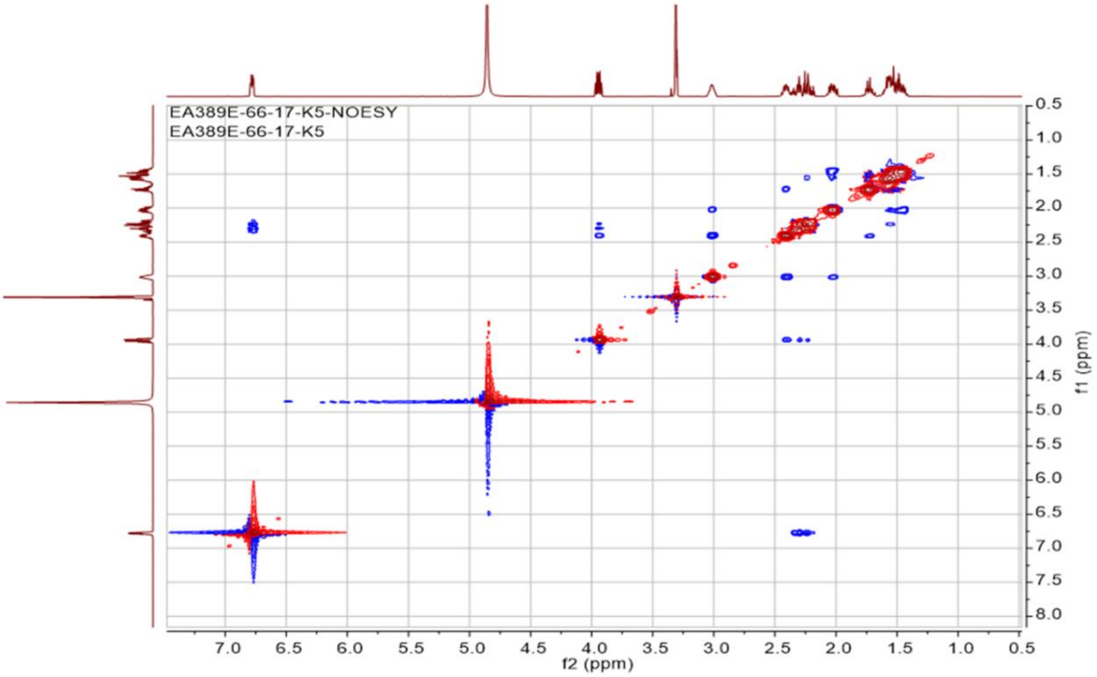

Figure S7. 1H-1H NOESY NMR spectrum of **1** (CD_3OD)

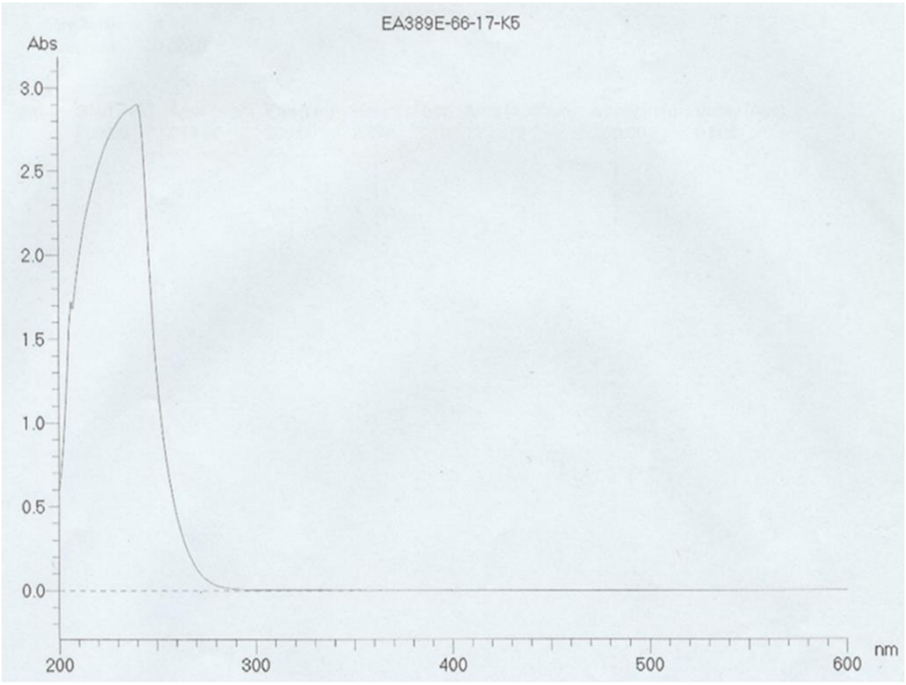

Figure S8. UV spectrum of **1**

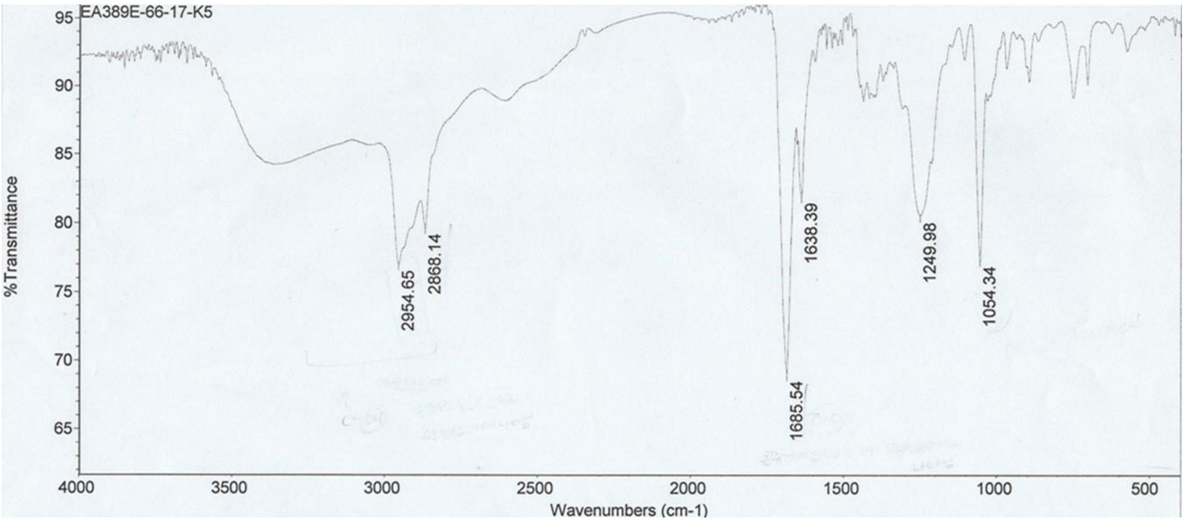

Figure S9. IR spectrum of **1**

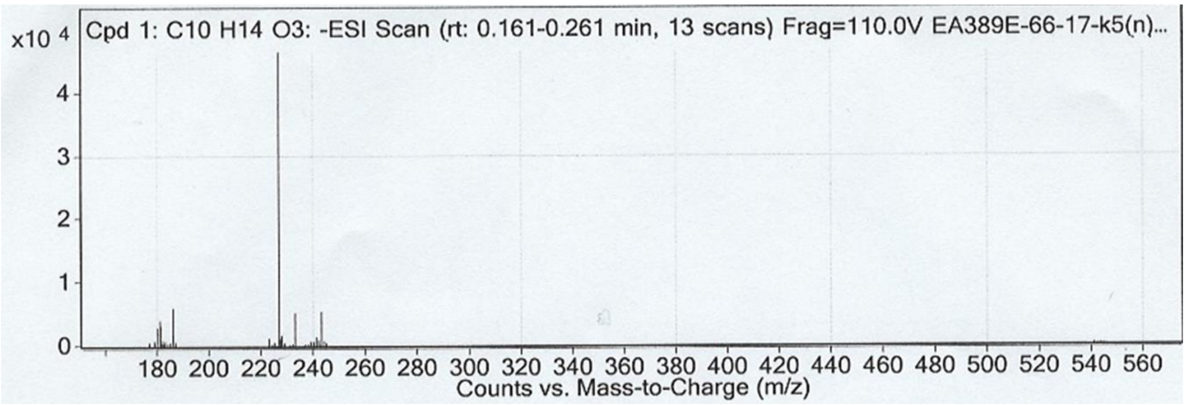

Figure S10. HRESIMS spectrum of **1**

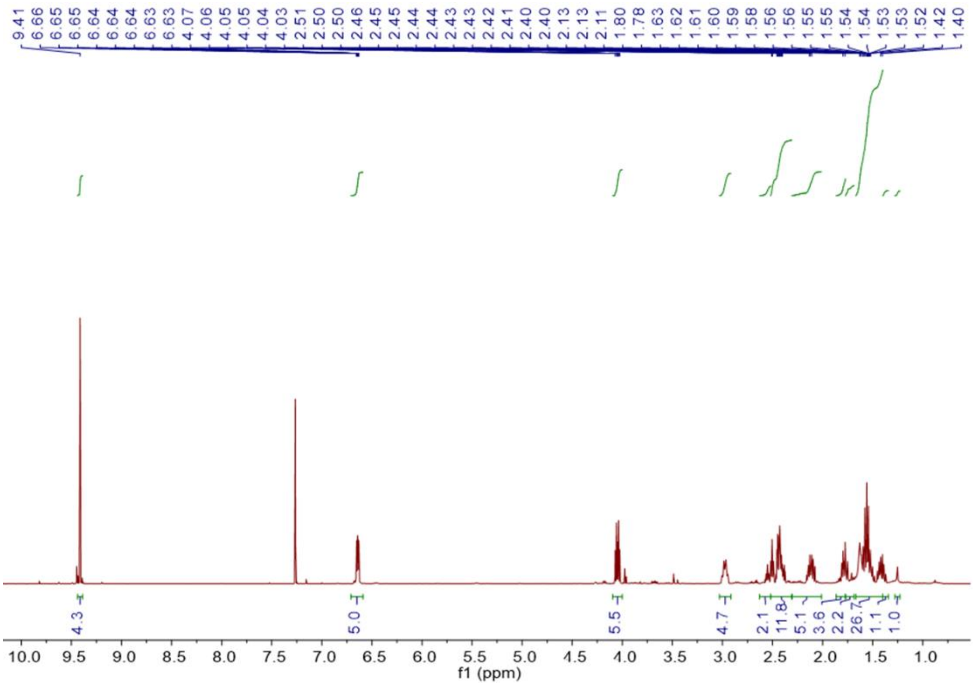

Figure S11. 1H NMR spectrum of **2** (400 MHz, $CDCl_3$)

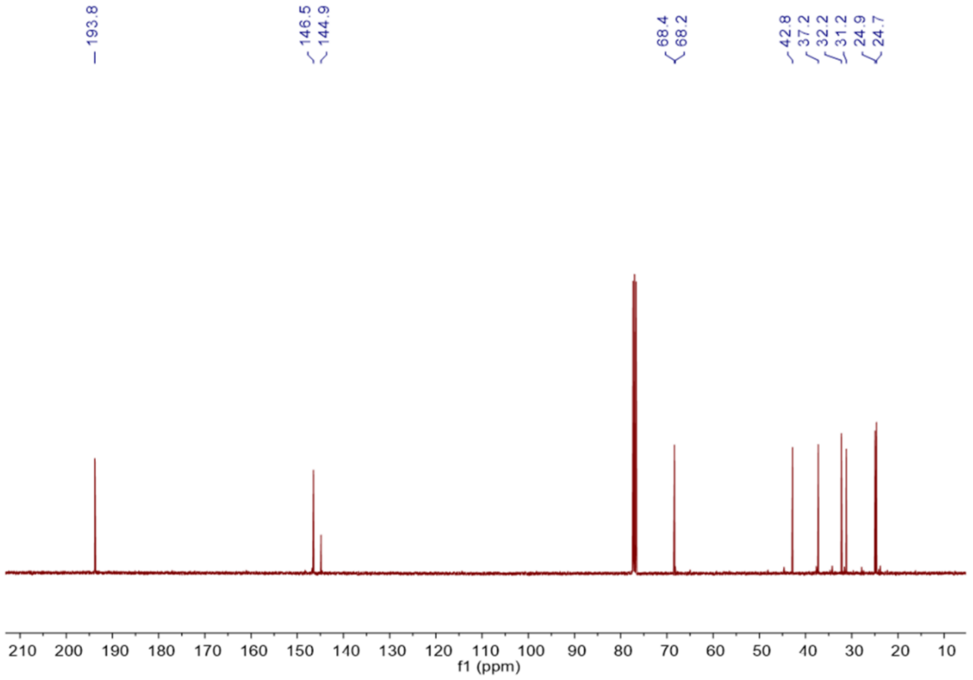

Figure S12. 13C NMR spectrum of **2** (100 MHz, $CDCl_3$)

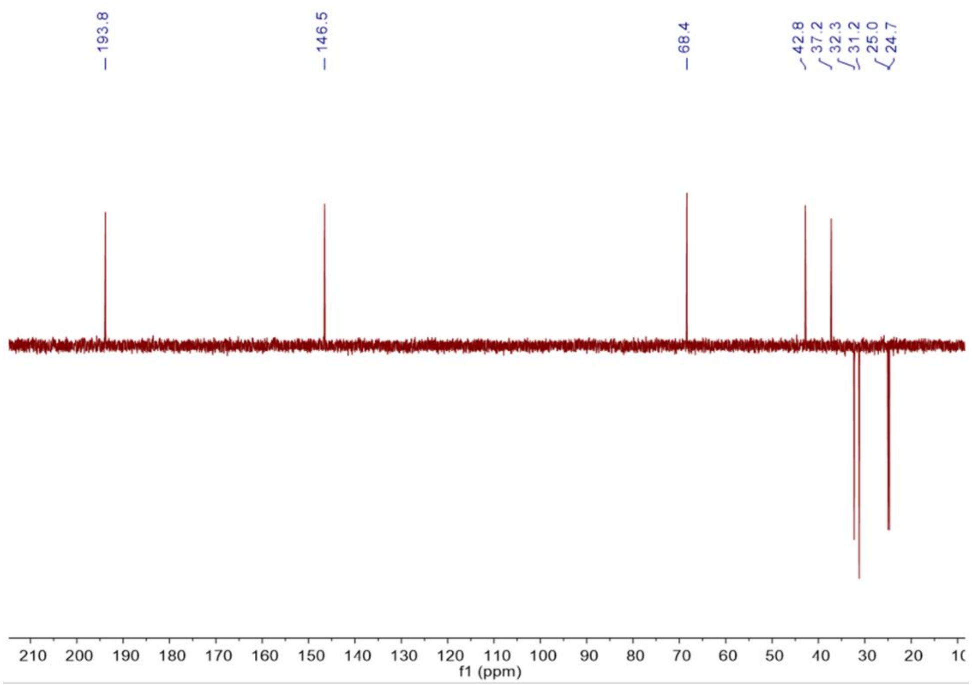

Figure S13. DEPT-135 NMR spectrum of **2** (100 MHz, $CDCl_3$)

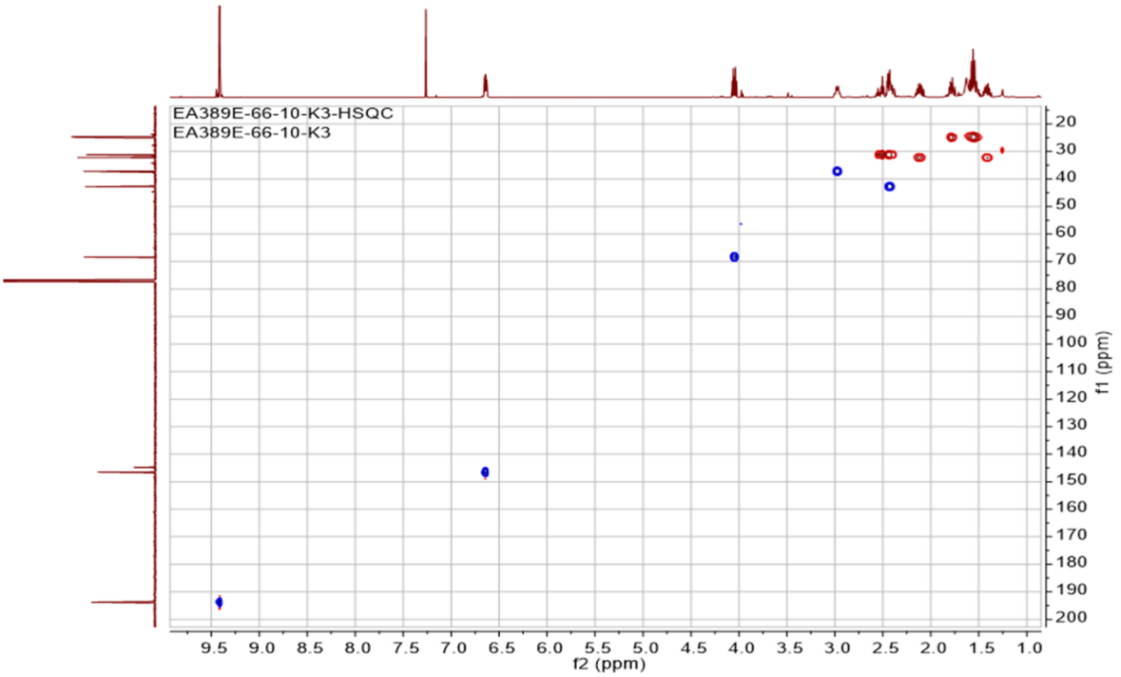

Figure S14. 1H-13C HSQC NMR spectrum of **2** ($CDCl_3$)

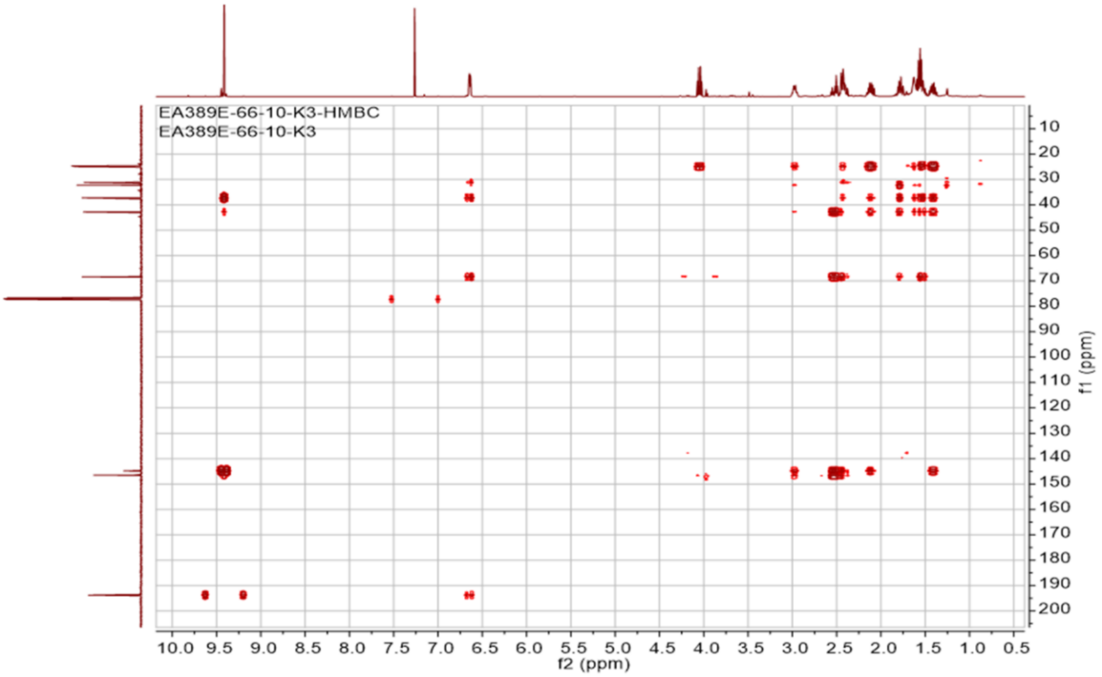

Figure S15. 1H-13C HMBC NMR spectrum of **2** ($CDCl_3$)

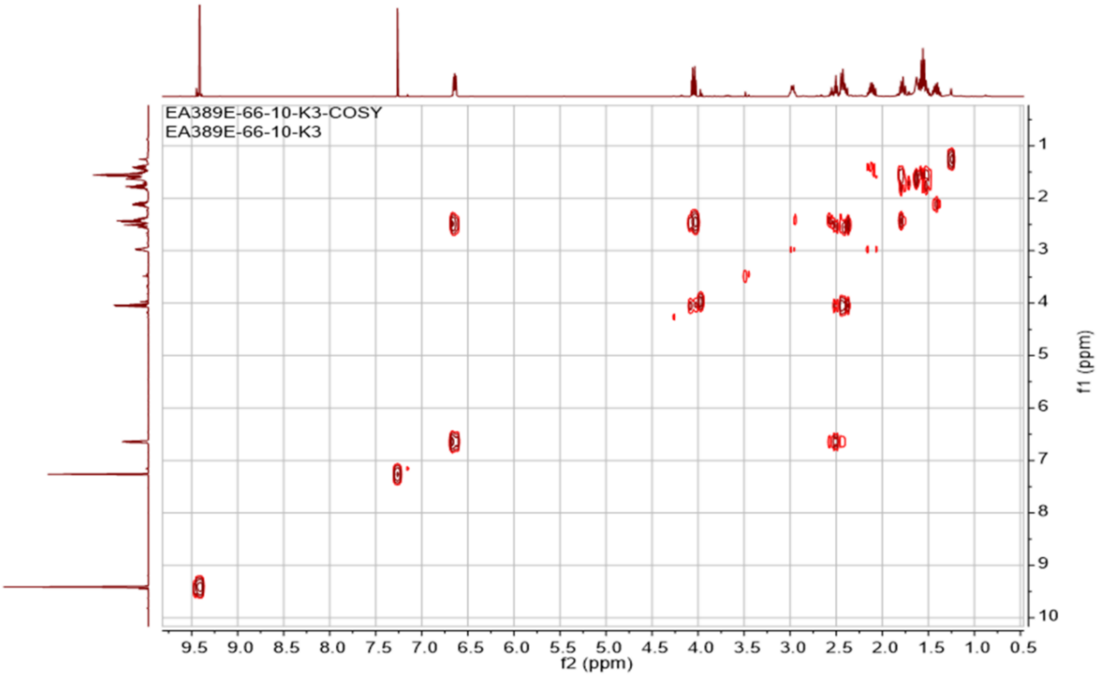

Figure S16. 1H-1H COSY NMR spectrum of **2** ($CDCl_3$)

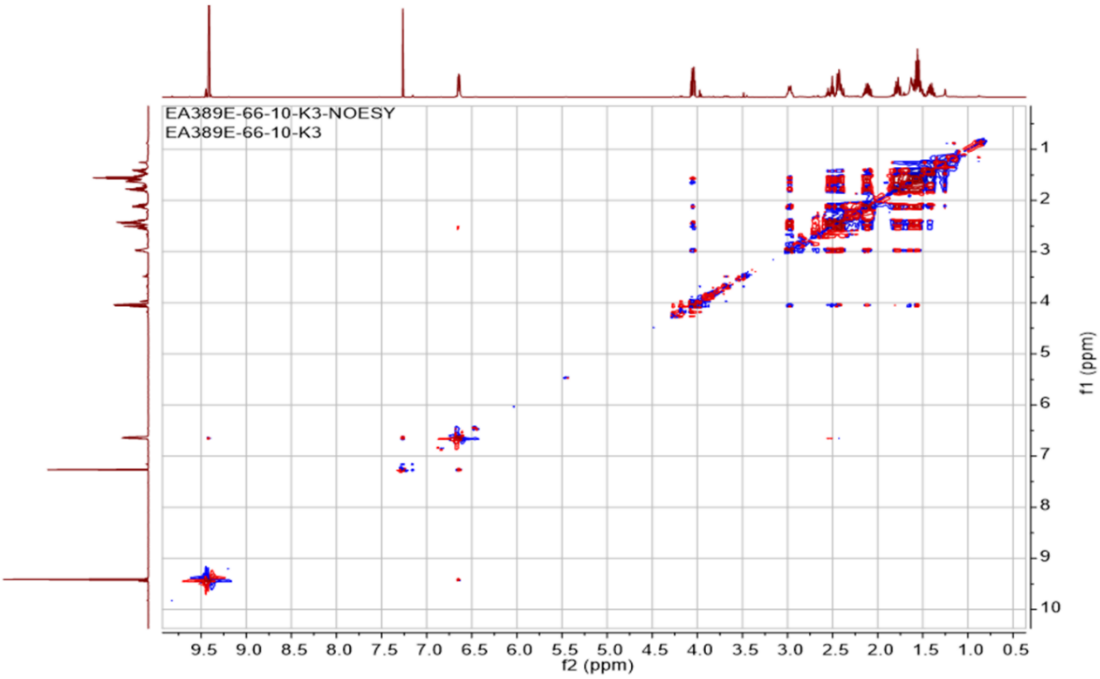

Figure S17. 1H-1H NOESY NMR spectrum of **2** ($CDCl_3$)

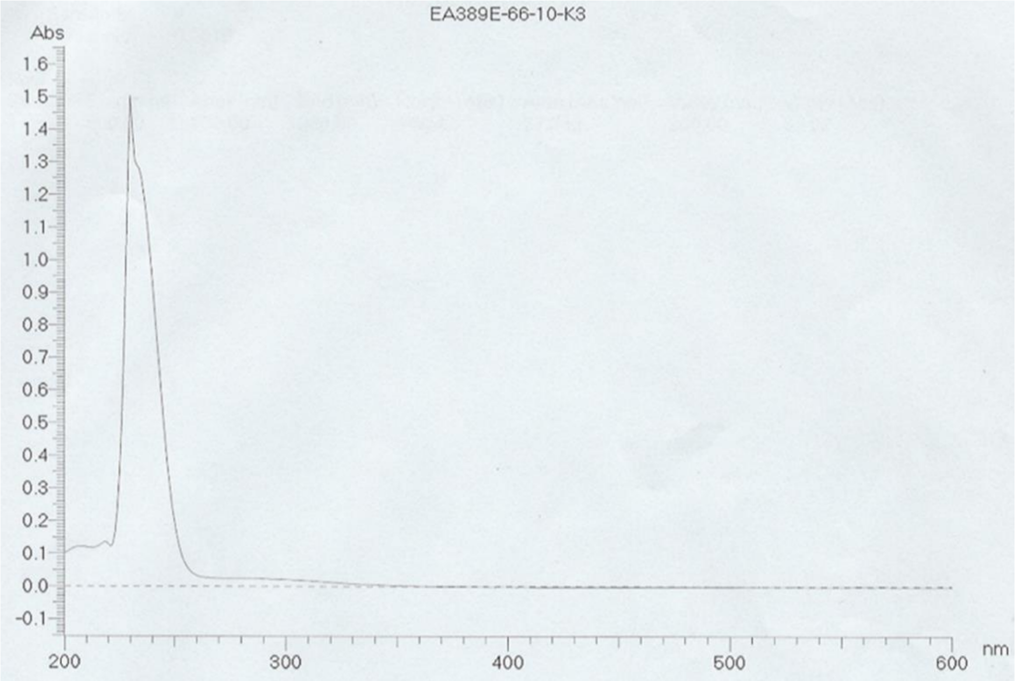

Figure S18. UV spectrum of **2**

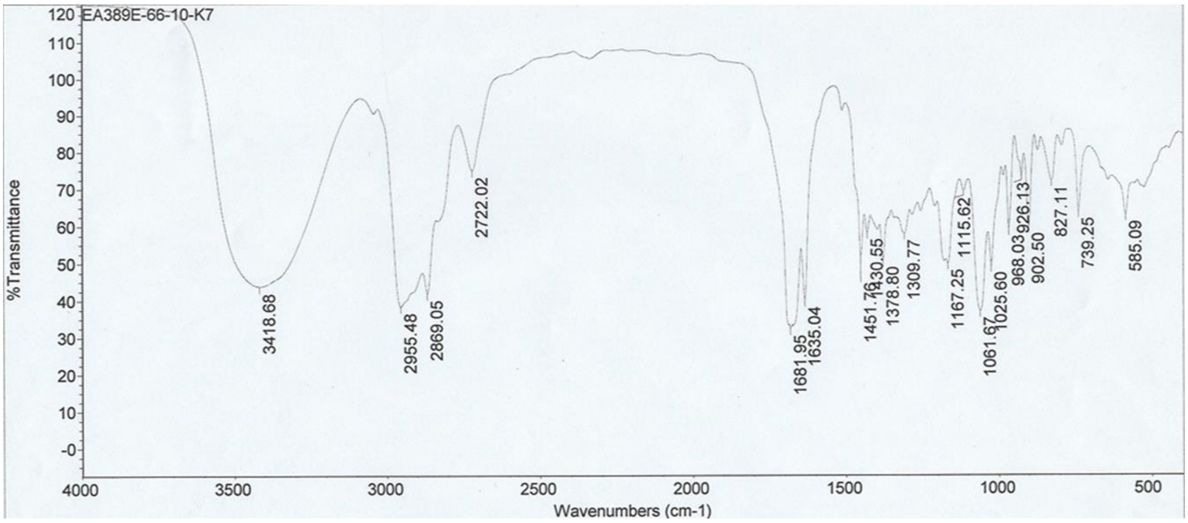

Figure S19. IR spectrum of **2**

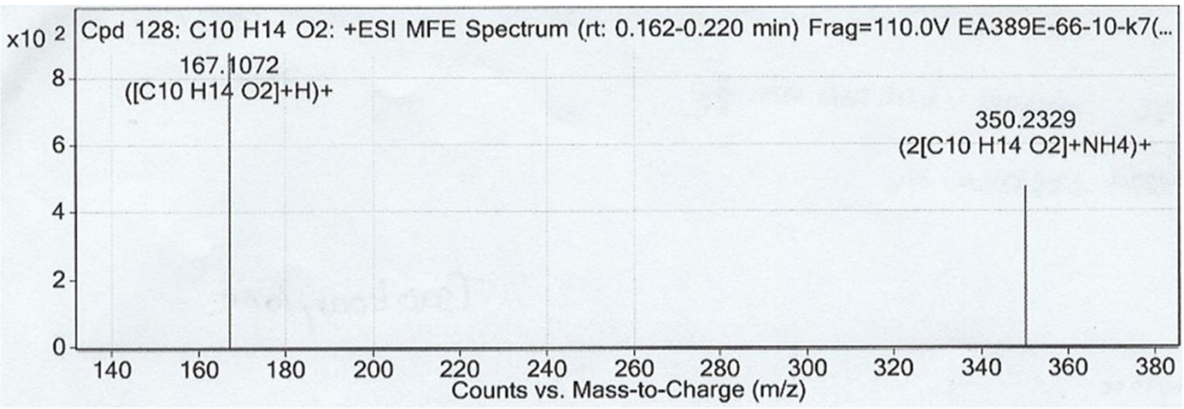

Figure S20. HRESIMS spectrum of **2**

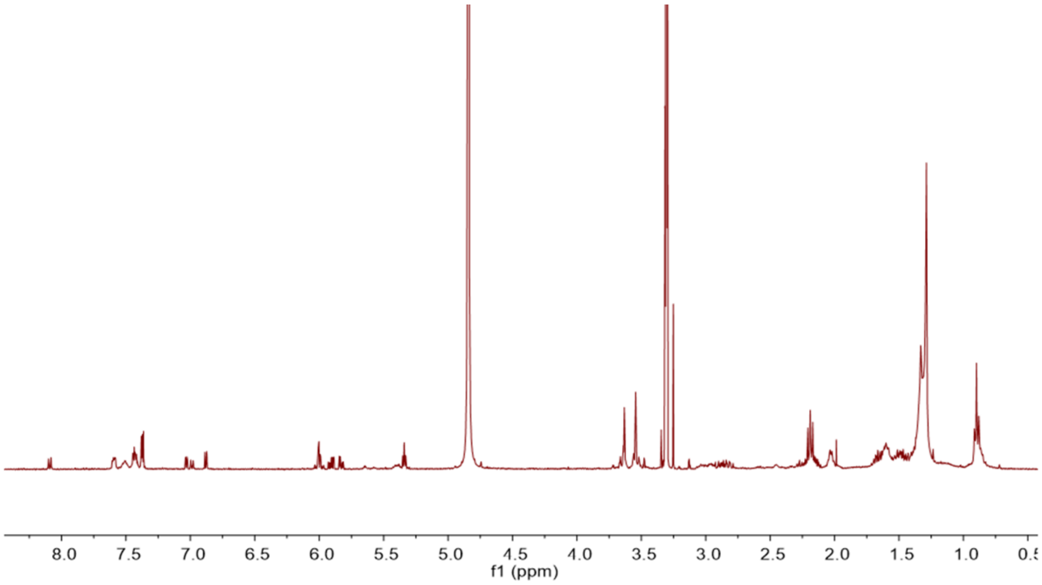

Figure S21. 1H -NMR (400 MHz, CD_3OD) of (R)-MTPA ester of **1**

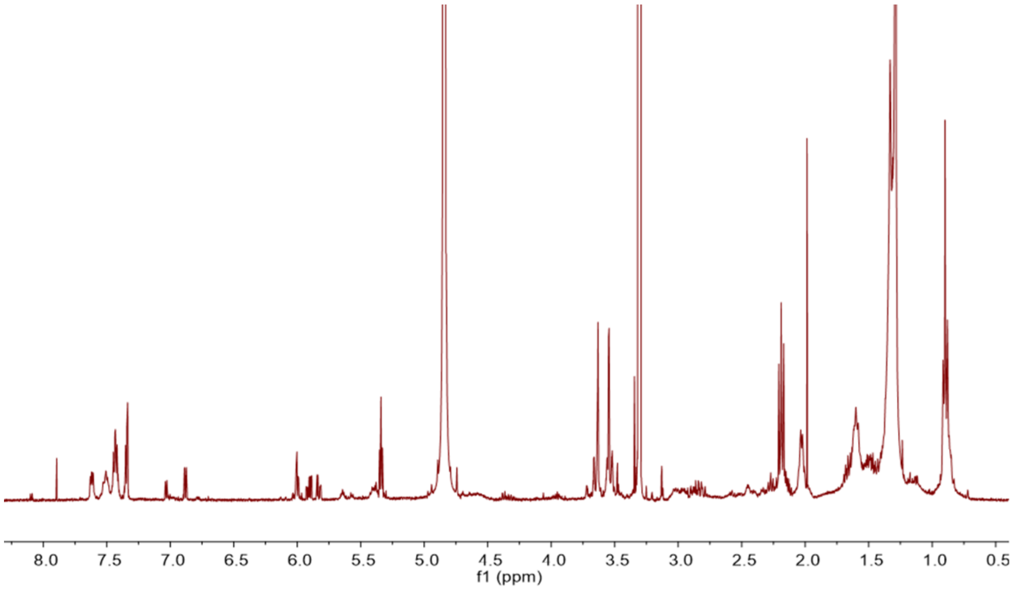

Figure S22. 1H -NMR (400 MHz, CD_3OD) of (S)-MTPA ester of **1**

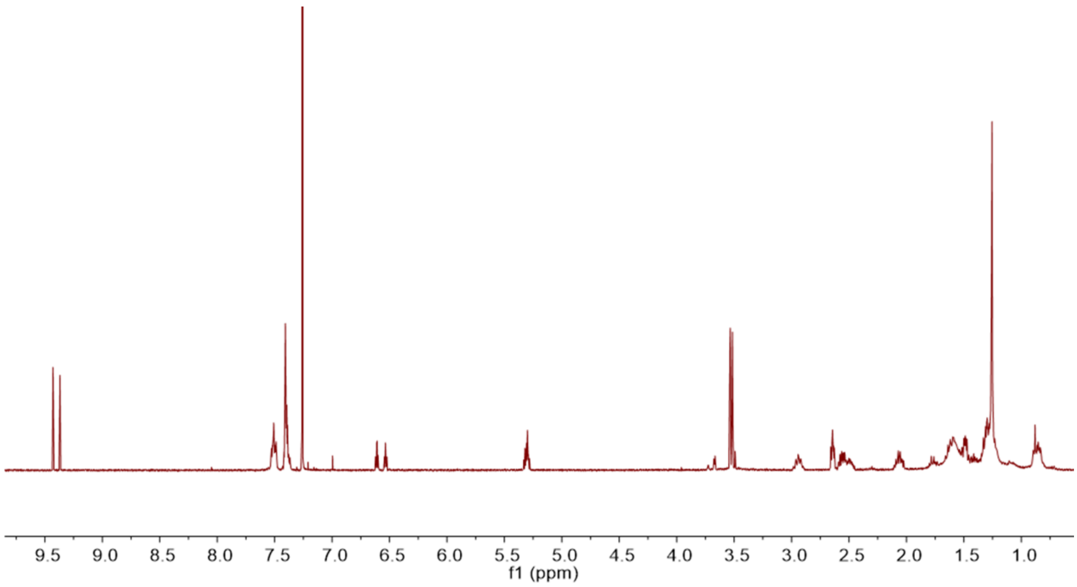

Figure S23. 1H -NMR (400 MHz, $CDCl_3$) of (R)-MTPA ester of **2**

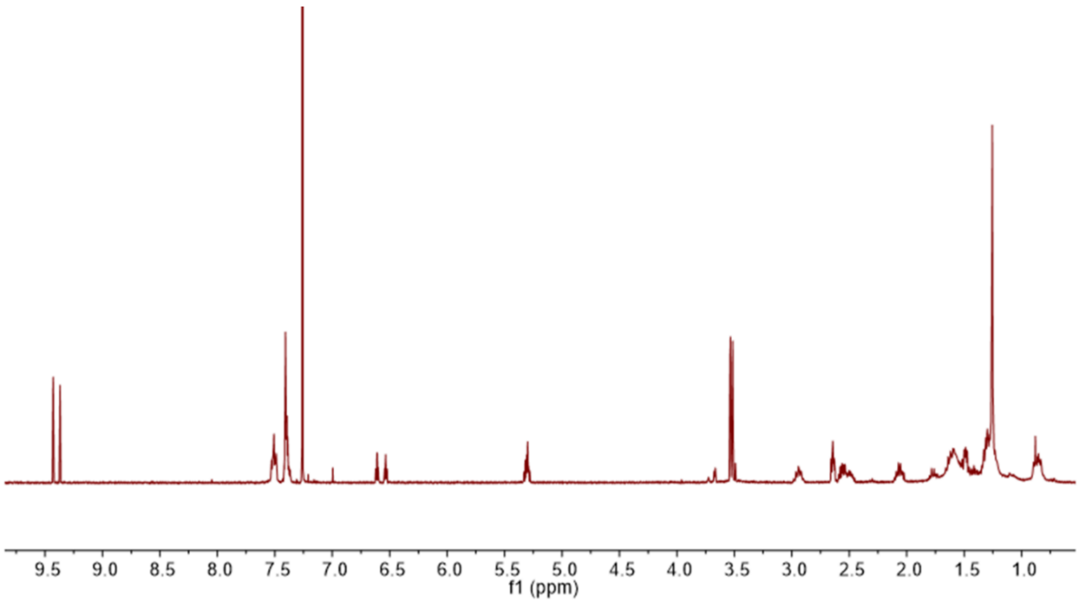

Figure S24. 1H -NMR (400 MHz, $CDCl_3$) of (S)-MTPA ester of **2**
